# Symptoms After COVID-19 Vaccination in Patients with Post-Acute Sequelae of SARS-CoV-2

**DOI:** 10.1007/s11606-022-07443-2

**Published:** 2022-02-22

**Authors:** Mayssam Nehme, Olivia Braillard, Julien Salamun, Frédérique Jacquerioz, Delphine S. Courvoisier, Hervé Spechbach, Idris Guessous

**Affiliations:** 1 Division of Primary Care Medicine, Geneva University Hospitals, Geneva, Switzerland; 2 Division of Tropical and Humanitarian Medicine, Geneva University Hospitals, Geneva, Switzerland; 3 Division of Infectious Diseases, Geneva University Hospitals, Geneva, Switzerland; 4 Cantonal Health Service, General Directorate for Health, Geneva, Switzerland; 5 Quality of Care Division, Medical Directorate, Geneva University Hospitals, Geneva, Switzerland; 6 Faculty of Medicine, University of Geneva, Geneva, Switzerland

## INTRODUCTION

While post-acute sequelae of SARS-CoV-2 are increasingly recognized,^1^ studies are still needed to identify risk factors, treatment options, and the duration and evolution of symptoms. Vaccination against SARS-CoV-2 prevents infection and acute complications. However, once infected, it is unclear whether vaccination can help prevent or improve long COVID or post-acute sequelae of SARS-CoV-2.^2,3^ In this study, we describe the association of vaccination and the evolution of six cardinal symptoms embodying post-acute sequelae of SARS-CoV-2.

## METHODS

From April 23 to July 27, 2021, an online survey was sent to 6987 individuals who previously tested positive for SARS-CoV-2 infection (RT-PCR test) at the outpatient testing center of the Geneva University Hospitals, Switzerland. The survey inquired about post-acute sequelae of SARS-CoV-2 and vaccination status. At the time of the study, the public health recommendations in Switzerland were for previously infected individuals to preferably receive one dose of vaccination only. Data was collected using REDCap v11.0.3 and analyzed using Stata, version 15.1 (StataCorp). The outcome of persistence of symptoms was defined as having any of the following: fatigue, difficulty concentrating or memory loss, loss of or change in smell, loss of or change in taste, shortness of breath, and headache. These six cardinal symptoms were chosen to characterize persistent symptoms post-SARS-CoV-2 based on previous studies.^1,4^

## RESULTS

Overall, 2094 participants answered the survey (response rate 29.9%). Of symptomatic participants, *n* = 1596 reported their symptoms developed after SARS-CoV-2 infection and that their comorbidities, when applicable, pre-dated the infection (characteristics in Table 1).

**Table 1:** Characteristics of Participants (*n* = 1596)

Age mean ± SD yr	43.5 ± 13.7
	*N* (%)
**Age categories**	
Below 40 yr	704 (44.1)
40–59 yr	697 (43.7)
60 yr and above	195 (12.2)
**Sex**	
Female	883 (55.3)
Male	713 (44.7)
**Time from infection to survey**	
3–6 months	33 (2.1)
6–9 months	1140 (71.4)
9–12 months	184 (11.5)
More than 12 months	239 (15.0)
**Smoking status**	
Never smoked	874 (54.8)
Current smoker	235 (14.7)
Ex-smoker	450 (28.2)
Prefer not to answer	37 (2.3)
**Comorbidities**	
None	858 (53.8)
Overweight	179 (11.2)
Sleeping disorder	122 (7.6)
Hypertension	111 (7.0)
Migraine	106 (6.6)
Anxiety	76 (4.8)
Other comorbidities	63 (3.9)
Depression	56 (3.5)
Other arthritis	56 (3.5)
Irritable bowel syndrome	54 (3.4)
Obesity	49 (3.1)
Respiratory disease	48 (3.0)
Tendinitis	47 (2.9)
Tension headache	44 (2.8)
Hypothyroidism	39 (2.4)
Cardiovascular disease	34 (2.1)
Anemia	33 (2.1)
Attention deficit	33 (2.1)
Diabetes	29 (1.8)
Other digestive disorder	27 (1.7)
Memory disorder	26 (1.6)
Other types of headache	24 (1.5)
Chronic fatigue	24 (1.5)
Immunosuppression	15 (0.9)
Dysmenorrhea	14 (0.9)
Hyperthyroidism	11 (0.7)
Chronic pain syndrome	10 (0.6)
Other neurological disorder	10 (0.6)
Rheumatoid arthritis	10 (0.6)
HIV	8 (0.5)
Thrombosis	8 (0.5)
Multiple sclerosis	6 (0.4)
Fibromyalgia	5 (0.3)
Crohn’s disease	5 (0.3)
Ulcerative colitis	5 (0.3)
Ankylosing spondylitis	5 (0.3)
Cancer	3 (0.2)
Renal disease	3 (0.2)
Other psychiatric disorders	3 (0.2)
Lupus	3 (0.2)
Reactive arthritis	2 (0.1)
Sjogren disease	1 (0.1)

*SD* standard deviation, *yr* year

Proportions of vaccinated participants included in our study were 47.1% (26.6% one dose, 20.5% two doses), compared to 65.3% of 228 asymptomatic participants (17.5% one dose, 47.8% two doses), and 69.0% of eligible adults in the general population by the end of July 2021 (18.8% one dose, 50.2% two doses).

**Fig. 1:**
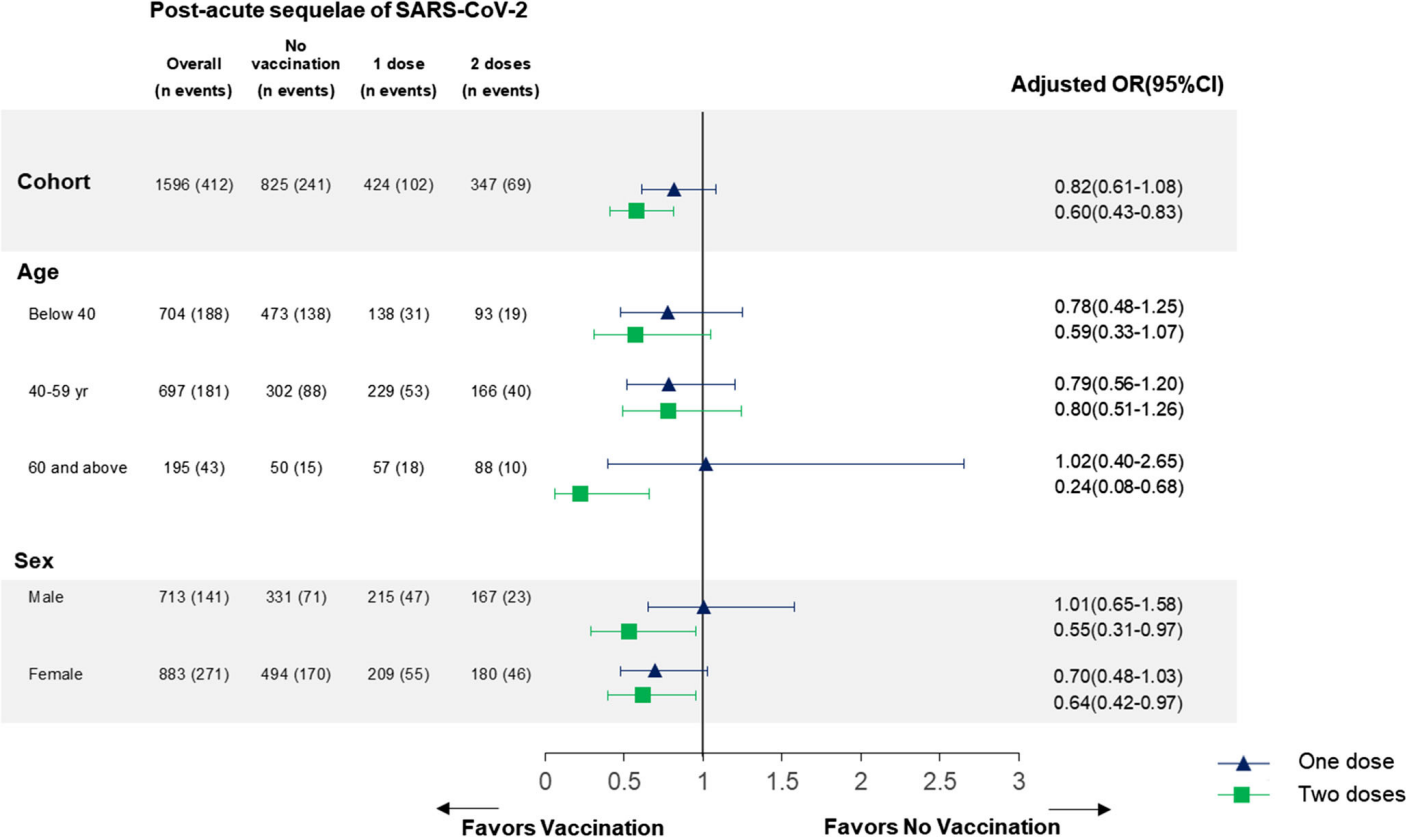
Associations between vaccination and the post-acute sequelae of SARS-CoV-2 infection, stratified by age groups and sex (*n* = 1596). Post-acute sequelae of SARS-CoV-2 were defined as the presence of fatigue, difficulty concentrating or memory loss, loss of or change in smell, loss of or change in taste, shortness of breath, and headache. These six cardinal symptoms were chosen to characterize persistent symptoms in long-COVID patients based on previous studies on the prevalence of symptoms more than 6 months after an infection.^1^ Participants answering (“Prefer not to answer”) were not included in this analysis (*n* = 8). Participants reporting never having symptoms were not included in this analysis of post-acute sequelae of SARS-CoV-2. Participants received the COVID-19 vaccine (mRNA-1273) of Moderna in 60.7% of cases, and the Comirnaty® (BNT162b2) vaccine of Pfizer/BioNTech in 38.5% of cases. Participants answered the survey on average 250.3 ± 72.1 days from their infection (257.8 ± 70.9 days for vaccinated *versus* 243.2 ± 72.5 days for non-vaccinated) and on average 40.3 ± 29.2 days after vaccination (range from 0 to 183 days, median 37 days, IQR 19–57). OR: odds ratios; CI: confidence interval. Odds ratios were adjusted for time for infection, age, sex, smoking, and the following comorbidities present prior to the infection: overweight or obese, hypertension, respiratory disease, cardiovascular disease, diabetes, migraine, tension headache, sleeping disorder, anxiety, depression, hypothyroidism, anemia, and chronic fatigue.

Following vaccination, symptoms disappeared (30.8%) or improved (4.7%) in 35.5% of cases, were stable in 28.7% of cases, and worsened in 3.3% of cases. Symptoms’ evolution was reported as other in 29.0% of cases and 2.6% preferred not to answer. Symptoms’ improvement or worsening occurred within 5 days post-vaccination in 69.6% and 82.3% of cases respectively. Vaccination (one or two doses) was associated with a decreased prevalence of the six cardinal post-SARS-CoV-2 symptoms (adjusted odds ratio, aOR 0.72; 0.56–0.92). Vaccination with 2 doses was associated with a decreased prevalence of dyspnea (aOR 0.34; 0.14–0.82) and change in taste (0.38; 0.18–0.83) as well as a decreased prevalence of any one symptom (aOR 0.60; 0.43–0.83) (details in Fig. 1).

## DISCUSSION

Vaccination was associated with a decreased prevalence of post-acute sequelae of SARS-CoV-2 infection compared to no vaccination. A recent study^2^ had shown similar results on a smaller scale (*n* = 36), and a pre-print with 900 participants^3^ showed improvement in 56.7% of cases post-vaccination, *versus* worsening (18.7%) and stability (24.6%). Another pre-print with 545 infected individuals showed that vaccination lowered symptom severity at 120 days.^5^ Postulated hypotheses include the potential correction of dysregulated immune or inflammatory responses, or the possible elimination of persisting viruses or viral remnants of SARS-CoV-2.^6^

Our study includes several limitations. It is challenging to attribute symptom improvement to vaccination *versus* other concomitant factors, with a potential indication bias if more symptomatic individuals preferred to withhold vaccination. To mitigate this potential bias, we adjusted for comorbidities and included only individuals with new symptoms post-infection. Also, while we cannot formally exclude indication bias, and specific new symptoms post-vaccination, we found no association between having symptoms prior to vaccination (at the time of testing) and vaccination rates. This suggests that more symptomatic participants were not more likely to get vaccinated (data not shown). Vaccinated participants also had a slightly longer time interval between the infection and the survey (14 days on average), mitigated by adjusting for time from infection. Finally, while our study is to date one of the largest, it lacked power for some stratified analyses, such as the impact of one vaccination dose. While acknowledging these limitations, we believe that the strength of the association, as well as a dose-response effect, could support a causal association, in addition to possible biological rationales evoked recently.^6^ If confirmed, this would mean that vaccination not only prevents infection but also can potentially improve post-acute sequelae of SARS-CoV-2.
